# A Novel Hybrid NN-ABPE-Based Calibration Method for Improving Accuracy of Lateration Positioning System

**DOI:** 10.3390/s21248204

**Published:** 2021-12-08

**Authors:** Milica Petrović, Maciej Ciężkowski, Sławomir Romaniuk, Adam Wolniakowski, Zoran Miljković

**Affiliations:** 1 Faculty of Mechanical Engineering, University of Belgrade, 11120 Belgrade, Serbia; zmiljkovic@mas.bg.ac.rs; 2 Faculty of Electrical Engineering, Białystok University of Technology, 15-351 Białystok, Poland; m.ciezkowski@pb.edu.pl (M.C.); s.romaniuk@pb.edu.pl (S.R.); a.wolniakowski@pb.edu.pl (A.W.)

**Keywords:** neural networks, Apparent Beacon Position Estimation, positioning systems, calibration, ultra wide band, non line of sight

## Abstract

Positioning systems based on the lateration method utilize distance measurements and the knowledge of the location of the beacons to estimate the position of the target object. Although most of the global positioning techniques rely on beacons whose locations are known *a priori*, miscellaneous factors and disturbances such as obstacles, reflections, signal propagation speed, the orientation of antennas, measurement offsets of the beacons hardware, electromagnetic noise, or delays can affect the measurement accuracy. In this paper, we propose a novel hybrid calibration method based on Neural Networks (NN) and Apparent Beacon Position Estimation (ABPE) to improve the accuracy of a lateration positioning system. The main idea of the proposed method is to use a two-step position correction pipeline that first performs the ABPE step to estimate the perceived positions of the beacons that are used in the standard position estimation algorithm and then corrects these initial estimates by filtering them with a multi-layer feed-forward neural network in the second step. In order to find an optimal neural network, 16 NN architectures with 10 learning algorithms and 12 different activation functions for hidden layers were implemented and tested in the MATLAB environment. The best training outcomes for NNs were then employed in two real-world indoor scenarios: without and with obstacles. With the aim to validate the proposed methodology in a scenario where a fast set-up of the system is desired, we tested eight different uniform sampling patterns to establish the influence of the number of the training samples on the accuracy of the system. The experimental results show that the proposed hybrid NN-ABPE method can achieve a high level of accuracy even in scenarios when a small number of calibration reference points are measured.

## 1. Introduction

One of the founding principles in the Industry 4.0 paradigm is the emphasis on the autonomy of the agents participating in the technological process. In order to achieve the autonomy of the mobile agents, e.g., Automated Guided Vehicles (AGVs) or mobile robots, it is paramount to provide a source of reliable navigational data, such as the information about the position of the agent or the layout of the environment it is working in. The former is handled by employing various types of positioning systems.

Accurate, precise, and reliable navigational data are especially important in applications where a given AGV has to cooperate with another vehicle or object, e.g., warehouse inventory inspection [1], cargo carriage in storehouse [2], or autonomous picking and palletizing [3]. Another example of an implementation of the reliable positioning system that is worth mentioning is customer navigation in a retail shop [4]. Demesure et al. [5] presents a navigation approach of mobile agents in the AGV-based manufacturing system. Sprunk et al. [6] propose a complex navigational system for the omnidirectional robot to implement enhanced logistic technology in industrial environment applications.

Different absolute positioning systems exist that use miscellaneous operating principles, such as Time-Of-Flight (TOF), Time Difference Of Arrival (TDOA), Phase Of Arrival (POA), or Received Signal Strength Indicator (RSSI) of a signal [7,8]; however, these can be generally divided according to the basic calculation principle into those using lateration and angulation-based techniques. The former utilizes the distance measurements between a set of reference points (often referred to as anchors or beacons) and the tracked object, while the latter relies on the angles measured between the object and the beacons. Nevertheless, measurements made in the real world are subject to noise coming from various sources. This noise, being propagated through the position estimation algorithm results in inaccurate estimates, thus limiting the usefulness of the system; therefore, it is essential to establish a procedure to mitigate the effect of the measurement noise on the output of the positioning system.

In our research, we utilize an Ultra Wide Band (UWB) technology-based positioning system due to its high applicability and current popularity for indoor applications. Moreover, the UWB rangefinder modules can be characterized by high accuracy (∼10 cm), ability to propagate signal through thin, non-metallic obstacles, and satisfactory range in an indoor environment [9,10]; however, this technology, similarly to other TOF-based ranging technologies, is highly sensitive to Non-Line-Of-Sight (NLOS) measurements. Radio signal traveling through any medium that is not a vacuum has an extended propagation time due to lower speed, which results in an overestimated distance measurement. Moreover, there can be different anchor-specific bias errors, caused by antenna misalignment, clock bias, wear of electronic parts, etc. [11]. Having that in mind, in the last two decades extensive research has been carried out in the field of lateral positioning systems, primarily focused on the development of algorithms used for the minimization of the estimated positioning error.

Sinriech et al. [12] analyzed configuration sensitivity of landmark navigation methods to improve the accuracy of AGV-based material handling systems operating in an industrial environment. Experimental results, including simulations of the static and dynamic performance of the vehicle, indicate that the triangulation positioning system is sensitive to noisy data as well as to different landmark and vehicle configurations.

Loevsky and Shimshoni [13] proposed another efficient landmark-based system for indoor localization of mobile robots and AGVs: based on the efficient triangulation method and sensors for bearing measurements of different landmarks, the proposed localization system enables a mobile robot to be accurately localized in motion and eliminate misidentified landmarks.

Aksu et al. [14] proposed a neural-network-based method to estimate the location of Bluetooth-enabled devices. The multi-layer perceptron network model with a back-propagation learning algorithm was applied to predict 2D coordinate location according to Received Signal Strength Information (RSSI) collected by three Bluetooth USB Adapters (BUAs). Although the authors made conclusions related to the effect of training sets inside and outside of the triangle formed by the three BUAs, they did not analyze the effects of different neural network architectures and learning algorithms on the estimation accuracy as well as how the number of training samples affects the set-up time.

Pelka et al. [15] developed an iterative algorithm, which was applied to determine the anchor position according to available distance measurements between anchors. Although the position problem is solved with the mean error of 0.62 m and without requirements for GPS data or prior knowledge, simulation results indicate that the precision of the distance measurements significantly affects the outcome of the algorithm.

We can consider an example of the beacon-based systems for angle measurement in mobile robotic applications [16]. This low power and flexible solution for robot positioning is named BeAMS, requires only one communication channel, is used for angle measurement and beacon identification. The authors proposed the mechanical design of the sensor as well as the theoretical analysis of the errors of the measured angles. The proposed model was compared with simulated and real measurements, and the achieved final error is lower than 0.24${}^{\circ }$.

Meissner et al. [17] proposed an indoor positioning algorithm based on the UWB signal and the *a priori* given floor plan information. The robust and accurate indoor localization was achieved with a receiver that uses single and double reflections of the transmitted signal in the room walls. According to *a priori* known room geometry, the measured reflections of the transmitted signal are mapped to virtual anchors with known positions and further used to estimate the unknown position of the receiver. Although the authors proposed the scheme for mapping measurements to the virtual anchors, they did not investigate the influence of the number of calibration reference points to achieve a short set-up procedure time.

Soltani et al. [18] conducted research to improve the Cluster-based Movable Tag Localization (CMTL) [19]. They proposed a localization method based on a Radio Frequency Identification (RFID) system for localization of the resources and used neural networks to overcome the limitations of empirical weighted averaging formulas. The proposed method forms a grid of virtual reference tags within the selected cluster of real reference tags and uses neural networks to obtain the position of the target tag. However, the authors have not considered the NN architectures with more than one hidden layer, as well as the impact of different learning algorithms and activation functions on the localization accuracy of the target tag.

Another example of the optimization of the positioning system is the use of additional calibration modules with a different number of calibration units to improve the average-position error in the 3D real-time localization system [20]. Three localization methods were used in the proposed research. The optimal configuration of calibration units was obtained in a simulation and tested in two real-world experiments. The small number of calibration units provides the best improvement-to-cost ratio; however, the most significant improvement of the average-position error is in the Z (vertical) direction; therefore, the proposed system is not recommended for 2D lateral positioning systems.

In our previous work, we proposed a method for improving the accuracy of the static infrared (IR) triangulation positioning system and eliminating errors caused by signal disturbances (e.g., reflections and multipathing) and/or inaccurate determination of the position of the beacons [21]. The presented methodology uses beacon–receiver angles that are measured by the receiver being placed at the known reference points and thereafter estimates the apparent beacon positions. The main advantage of the proposed methodology is that the *a priori* information about the locations of the beacons is not required. In further research, we developed a calibration method for a lateration positioning system based on measuring beacon–receiver distances. According to measurements based on lateration and known reference positions of the receivers, the proposed method estimates unknown positions of the beacons (i.e., apparent positions) and compensates for the static errors [22].

Compared to the previously reported state-of-the-art methods, the major contributions of the paper can be summarized as follows:

- A new hybrid procedure based on ABPE and NNs is used to correct the positioning system measurements;
- Different neural network architectures are employed in order to find the optimally tuned parameters for the proposed calibration problem, e.g., 16 neural network architectures with 10 learning algorithms and 12 different activation functions for hidden layers are trained and validated in MATLAB environment to learn and predict measured positions;
- The performance of the novel hybrid NN-ABPE-based method in terms of both the set-up time and accuracy is compared to the state-of-the-art calibration methods, i.e., mapping with a distortion model, Bias and Scale Factor Estimation (BSFE), and Apparent Beacon Position Estimation (ABPE). Experimental results obtained in two different scenarios (environment with and without obstacles) confirmed the effectiveness of the proposed methodology to predict positioning system measurement errors in real-world situations.

## 2. Methods

Let us consider a lateration-based local positioning system, where the positions of beacons/anchors are known, and the position of the tag/receiver has to be determined. In order to find the receiver position, the beacon–receiver distance measurements ($d_{i}$ in Equation (1) and the known beacon positions ($X_{i},Y_{i}$) are used to define a cost function described by the Equation (1) which can be minimized with respect to unknown receiver position (x,y) with the Nonlinear Least Squares (NLS) method.

(1) $$\underset{(x,y)\in R}{\arg\min}\;\;\sum_{i=0}^{m}{\left(d_{i}-\sqrt{{\left(X_{i}-x\right)}^{2}+{\left(Y_{i}-y\right)}^{2}}\right)}^{2}$$

More on the lateration method and NLS itself can be found in [23,24].

### 2.1. Position Correction in Positioning Systems

The position estimated directly through the Least Squares Method is prone to be inaccurate due to the influence of various disturbing factors. In order to improve the accuracy of the system, this initial estimate has to be corrected.

One of the common correction methods is to assume that the accuracy of the position estimate can be improved by applying a correction function to the original estimate (2):

(2) $$\begin{array}{c} r^{*}=C\left(r,F\right) \end{array}$$

where $r^{*}={\left[x^{*},\;y^{*}\right]}^{T}$ denotes the improved position estimate, *C* is the correction function, $r={\left[x,\;y\right]}^{T}$ is the original estimate, and F is a set of correction function parameters. The selection of the correction function *C* and the learning of its parameters *F* is called the *calibration procedure*. Typically, the calibration involves the collection of a number of sample position measurements $R_{m}=\left[r_{m1},\;r_{m2},\;\cdots ,\;r_{mn}\right]$ obtained through the positioning system and a matching number of the ground-truth positions $R_{r}=\left[r_{r1},\;r_{r2},\;\cdots ,\;r_{rn}\right]$ obtained through a reference system. The correction function parameters F are then learned based on the relations between the sets $R_{m}$ and $R_{r}$. Once the calibration procedure is completed during the set-up of the system, subsequent position measurements can be corrected online during the system operation with little overhead. Because of the initial set-up cost associated with learning the correction function *C*, it is desirable to select a function that can be robustly trained on the least amount of training samples possible.

#### 2.1.1. Distortion Model

A simple and common approach, derived from the image rectification procedure in vision [25,26], is to assume a distortion model in the form of a quadratic mapping between the original position estimate $r={\left[x,\;y\right]}^{T}$ and the corrected estimate $r^{*}={\left[x^{*},\;y^{*}\right]}^{T}$, where lower-case *x*, *y*, and $x^{*}$, $y^{*}$ denote the original and corrected coordinates respectively. Such mapping can be defined as (3) and (4):

(3) $$\begin{array}{c} x^{*}=f_{11}x^{2}+f_{12}xy+f_{13}y^{2}+f_{14}x+f_{15}y+f_{16} \end{array}$$

(4) $$\begin{array}{c} y^{*}=f_{21}x^{2}+f_{22}xy+f_{23}y^{2}+f_{24}x+f_{25}y+f_{26} \end{array}$$

which can be conveniently expressed for simultaneous transformation of multiple original estimates $R_{2xn}=\left[r_{1},\;r_{2},\;\cdots ,\;r_{n}\right]$, where $r_{i}={\left[x_{i},\;y_{i}\right]}^{T}$, into corrected estimates $R_{2xn}^{*}=\left[r_{1}^{*},\;r_{2}^{*},\;\cdots ,\;r_{n}^{*}\right]$ in the matrix form (5):

(5) $$\begin{array}{c} R_{2xn}^{*}=C\left(R_{2xn},F_{2x6}\right)=F_{2x6}\cdot M_{6xn}\left(R\right) \end{array}$$

where matrix $M_{6xn}\left(R\right)$ is constructed as (6):

(6) $$\begin{array}{c} M_{6xn}\left(R\right)=\left[\begin{array}{cccc} x_{1}^{2} & x_{2}^{2} & \cdots & x_{n}^{2}\\ x_{1}y_{1} & x_{2}y_{2} & \cdots & x_{n}y_{n}\\ y_{1}^{2} & y_{2}^{2} & \cdots & y_{n}^{2}\\ x_{1} & x_{2} & \cdots & x_{n}\\ y_{1} & y_{2} & \cdots & y_{n}\\ 1 & 1 & \cdots & 1 \end{array}\right] \end{array}$$

The units of $f_{ij}$ coefficients in the matrix F are chosen such that the units in Equations (3) and (4) match. For example, the coefficient $f_{11}$ which is multiplied by *x* coordinate squared, has a unit of [$m^{-1}$]. In general, the coefficients $f_{ij}$ have the following units: [$m^{-1}$] for j∈{1,2,3}, [1] for j∈{4,5} and [m] for j=6.

The quadratic distortion model involves 12 independent parameters that may be learned from n=6 sample measurements and the matching reference positions using the following Equation (7):

(7) $$\begin{array}{c} F_{2x6}=R_{r2x6}\cdot M_{6x6}{\left(R_{m}\right)}^{-1} \end{array}$$

It should be noted that matrix M may not always be invertible. Six measurements are required to obtain the exact solution, but typically a larger data set is collected in order to achieve better correction accuracy and thus the M matrix is no longer square. In that case, the F matrix is obtained in a more general way that satisfies the least-square relationship between the $M(R_{r})$ and $M{\left(R_{m}\right)}^{-1}$, (8):

(8) $$\begin{array}{c} F_{2x6}=R_{r2xn}\cdot M_{6xn}{\left(R_{m}\right)}^{+} \end{array}$$

where $M{\left(R_{m}\right)}^{+}$ denotes the Moore–Penrose pseudo-inverse of the $M(R_{m})$ matrix.

This method is referred to as DQM (Distortion Quadratic Model) in further text.

#### 2.1.2. Apparent Beacon Position Estimation

The most common approach to calculate the unknown receiver position in the lateration system, as was mentioned in the introduction to Section 2, is to utilize the measured receiver–beacon distances and known beacon positions in the local reference frame. This approach requires that the position of the beacons is accurately measured. The beacon–receiver distance measurements are made via radio waves channel and, unfortunately, are always error-prone. The sources of these errors may be the following: the orientation of radio antennas, measurement offsets of beacons hardware, electromagnetic noise, or some objects in the environment interfering with the measurement. These measurement errors cause the beacons to be “seen” by the receiver in a slightly different position than they actually are.

We have developed a method called the Apparent Beacon Position Estimation (ABPE) method that calculates these *apparent positions*. The great advantage of this method (apart from improving the receiver position estimation) is that there is no necessity to provide *a priori* measurements of the beacon positions—the ABPE method finds them itself. The method requires as input the positions of a number of reference points in a given local reference frame and distances between the beacons and the receiver placed in reference points. The positions of the reference points should be measured with an additional measurement system (e.g., with a measuring tape), while beacon–receiver distances are measured via a lateration positioning system (in our case it was the UWB positioning system).

The ABPE method utilizes the distance measurements between the beacons placed in unknown positions $A_{i}$ and the receiver that is placed in known reference points $P_{j}$ in order to determine these unknown beacons positions $A_{i}$. The following algorithm estimates apparent position of the beacons, i.e., what positions of the beacons are seen by the receivers.

**Figure 1:**
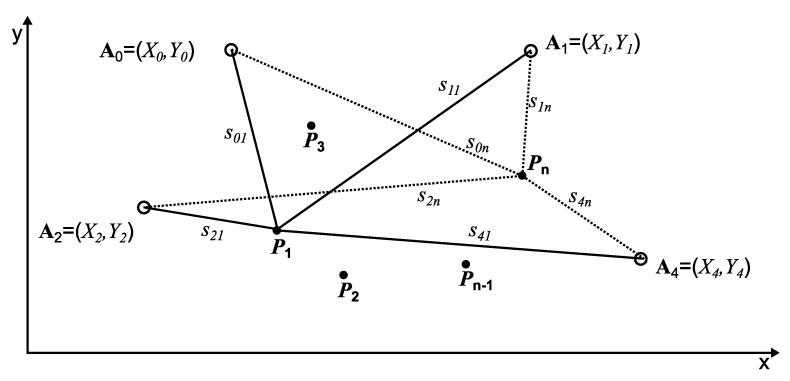
The ABPE method based on the reference points.

In the lateration-based positioning systems, we can distinguish the following Equation (9):

(9) $$\begin{array}{c} s_{ij}=\sqrt{{\left(X_{i}-x_{j}\right)}^{2}+{\left(Y_{i}-y_{j}\right)}^{2}} \end{array}$$

which describes the distance between the beacon $A_{i}=\left(X_{i},Y_{i}\right)$ and the reference point $P_{j}=\left(x_{j},y_{j}\right)$ (see Figure 1).

Since measurements are affected by numerous disturbances, the distance calculated in Equation (9) will never be equal to the beacon–receiver distance measurement in the real world when the receiver is placed in reference point $P_{j}$. To find the best fit of the measurement data (distance measurements via UWB) to the distances calculated by Equation (9), the following cost function should be minimized with respect to unknown beacons positions $A_{i}$ (10):

(10) $$\begin{array}{c} \begin{array}{c} \underset{(X_{i},Y_{i})\in R}{\arg\min}\;\;\sum_{j=1}^{n}\sum_{i=0}^{m}{\left(d_{ij}^{2}-s_{ij}^{2}\right)}^{2} \end{array} \end{array}$$

where $\left(X_{i},Y_{i}\right)$ are the apparent positions of the beacons and $d_{ij}$ correspond to the beacon–receiver distances measurements taken by the UWB receiver placed at points $P_{j}$. We refer the reader to [22] for a more detailed description of the algorithm.

#### 2.1.3. Bias and Scale Factor Estimation

The accuracy of the calculated positions by means of the lateration method strongly depends on the distances that are always measured with some noise. In the Bias and Scale Factor Estimation, it is assumed that distance measurements are disturbed by some bias and scale factor that are different for each of the anchors. The aforementioned assumptions can be summarized in the following Equation (11) describing the real distance:

(11) l=Sd+B

where *d* is the measured anchor-tag distance, *S* is an unknown scale factor and *B* is an unknown bias factor. From Equation (11) we can induce the real distance between anchor $A_{i}$ and the tag placed at position $P_{j}$, (12):

(12) $$l_{ij}=S_{i}d_{ij}+B_{i}$$

where $d_{ij}$ is the measured distance between anchor $A_{i}$ and tag placed in the reference point $P_{j}$. The parameters $S_{i}$, $B_{i}$ define, respectively, scale and bias factors for beacon *i*. Moreover, the real distances can also be calculated utilizing the knowledge on the position of anchors and tag (13):

(13) $$l_{ij}=\sqrt{{\left(X_{i}-x_{j}\right)}^{2}+{\left(Y_{i}-y_{j}\right)}^{2}}$$

where $A_{i}=\left(X_{i},Y_{i}\right)$ is the known coordinates of the anchors and $P_{j}=\left(x_{j},y_{j}\right)$ is the known coordinates of the tag placed in the reference point. Next, we can utilize Equations (12) and (13) in order to find the unknown parameters $S_{i}$ and $B_{i}$ by minimizing the following cost function (14):

(14) $$\begin{array}{c} \underset{(S_{0},B_{0},S_{1},B_{1},\ldots ,S_{m},B_{m})\in R}{\arg\min}\;\;\sum_{j=1}^{n}\sum_{i=0}^{m}{\left(l_{ij}^{2}-d_{ij}^{2}\right)}^{2} \end{array}$$

where $\left(S_{0},B_{0},S_{1},B_{1},\ldots ,S_{m},B_{m}\right)$ are the scale and bias factors of the measurements from subsequent *m* anchor. The Nelder-–Mead method can be used the solve the presented minimization function [27].

#### 2.1.4. Neural Networks

The neural networks represent a soft-computing paradigm of artificial intelligence defined as a connective model for reasoning based on an analogy with the biological neurological system. The connective model is composed of interconnected elements (aka neurons) that give the network the cognitive ability to learn and generalize acquired knowledge. The most widely used models are feed-forward neural networks with back-propagation algorithm (BP neural networks), which find wide application in solving prediction, classification, and approximation problems [28,29].

Generally, the BP network consists of neurons grouped in layers. In addition to the input and output layers, the network can have one or more hidden layers. Neurons receive input signals i.e., information from the environment or the other neurons, through connections with appropriate weight strength. Input data presented to the neural network through the input layer can be defined as matrix I (15):

(15) $$I=[x_{1},\;\ldots x_{i},\;\ldots ,x_{M}],\;$$

where *i* represents the *i*-th input vector, i=1,…,M. The weighted output of the *k*-th neuron in *l*-th layer is defined as (16):

(16) $$o_{ik}^{l}=\sum_{j=1}^{K_{l}}w_{kj}^{l}I_{ij}^{l}+\theta_{k}^{l},$$

where $K_{l}$ represents the total number of neurons in the previous layer, $j=1,\ldots ,K_{l}$, *l* represents number of layers, l=1,…,L, the weight strength between neurons *j* and *k* is defined as $w_{kj}^{l}$, and $\theta_{k}^{l}$ represent a bias value of the *k*-th neuron.

The output value of *k*-th neuron in *l*-th layer is calculated by applying the activation function $f_{ik}^{l}$ on weighted output $o_{ik}^{l}$, (17):

(17) $$O_{ik}^{l}=f_{ik}^{l}\left(o_{ik}^{l}\right)$$

The output of the network is the output of its final *L*-th layer and is denoted as:

(18) $$net\left(I,W,\Theta ,f\right)=O_{i}^{L}k$$

where **I** is the input data, **W** is the matrix of the weights, Θ is the matrix of bias values, and *f* is the vector of activation functions for consecutive layers.

The overall error between the actual and pre-defined output is calculated by Equation (19):

(19) $$E_{pi}=\frac{1}{2}\sum_{k=1}^{K_{L}}{\left(y_{ik}-O_{ik}^{L}\right)}^{2}$$

The cognitive ability of the BP neural network can be achieved through the supervised learning (training) process based on gradient descent method that modifies the weights between the neurons by applying different modification procedures. Those iterative procedures can be formalized in various learning algorithms [30]. Therefore, after the overall error is calculated according to Equation (19), that error is propagated backwards through the network layers in order to modify weights between the neurons. The learning process is performed with the goal of minimizing the error between the actual output and the pre-defined output of the network.

The neural networks have attracted a significant amount of attention in the research community particularly due to their wide range of applications and successful implementations in solving various complex problems. The main advantages of neural networks that make them suitable for solving such problems are related to their (i) capability of flexible nonlinear modeling between dependent and independent variables, (ii) strong adaptability, as well as their (ii) learning and massively parallel computing abilities [31].

This data-driven approach is developed based on the features presented by the data sets, which makes it suitable to process fuzzy, nonlinear, and noise-containing data without the need to design any mathematical models. On the other side, a very important characteristic of neural networks is their adaptive nature, where learning by example is very appealing in scenarios there is a little or incomplete understanding of the problem to be solved, but experimental data are available. Finally, their high computational power is based on a densely interconnected large set of adaptive processing units forming the topological structure for distributed parallel information processing.

The neural networks provide significant success and benefits in solving processing problems that require real-time operation and interpretation of relationships between variables in multidimensional spaces. Furthermore, they have been used in scenarios when information about certain phenomena is noisy, partial, unknown and/or their connections are incomplete. As a result, they have been successfully applied for classification and regression [28] and widely used for solving many problems in the last decades in the domain of intelligent material transport [32,33]. In the following section, a novel hybrid calibration method based on BP neural networks and Apparent Beacon Position Estimation (ABPE) is proposed to improve the accuracy of a lateration positioning system.

#### 2.1.5. Hybrid NN-ABPE Method

**Figure 2:**
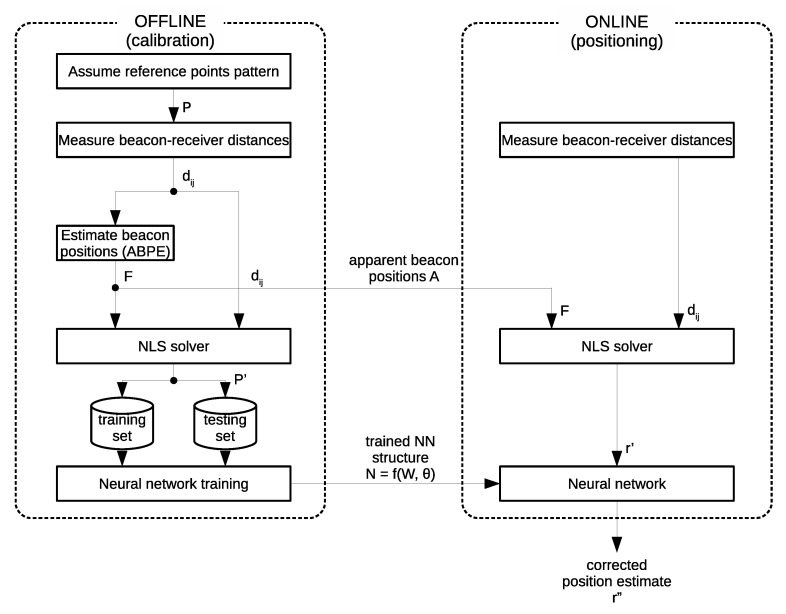
Diagram of the workflow of the hybrid NN-ABPE method.

In order to improve the performance of the position calibration methods, the benefits of apparent beacon estimation are synergized with advantages of neural networks to learn nonlinear mapping between experimentally acquired data. In this section, a novel hybrid calibration method developed for accurate prediction of measurement position and minimizing measurement errors is presented hereinafter. It consists of two stages: (1) the offline calibration stage in which input–output pairs for neural network training and the ABPE method training are collected, and (2) the online stage where the trained methods are used to estimate the target object position. The diagram of the workflow of the hybrid NN-ABPE method is presented in Figure 2.

Let us assume that the positioning system consists of *m* beacons with unknown positions $A_{i}=\left(X_{i},\;Y_{i}\right),\;i=1,\cdots ,m$.

**Offline stage.** The calibration stage starts with the equipment setup, where beacons are placed in arbitrary positions surrounding the workspace. Next step is the data collection phase, where the pattern $P_{j}=\left(x_{j},\;y_{j}\right),\;j=1,\cdots ,n$ of *n* reference points is assumed. The pattern P is chosen such that it uniformly covers the workspace with a desired resolution. The receiver is subsequently placed at consecutive points in the pattern. At each of these positions the beacon–receiver distances $d_{ij}$ are measured via UWB positioning system. The distances $d_{ij}$ and the pattern P are necessary as the input for the next stage of the algorithm.

The next step of the offline stage is to estimate the apparent beacon positions $A_{i}=\left(X_{i},\;Y_{i}\right),\;i=1,\cdots ,m$ using the ABPE method (see Section 2.1.2). Based on these estimated beacon positions and the measured beacon–receiver distances $d_{ij}$, the estimated positions of the reference points $P_{j}^{\prime }=\left(x_{j}^{\prime },\;y_{j}^{\prime }\right),\;j=1,\cdots ,n$ are calculated with a NLS solver (1). The estimated positions of the reference points $P^{\prime }$ are further used for neural network training. The neural network input is the reference points positions estimated via NLS: $P^{\prime }$, while the network is trained to output the ground-truth positions of the reference points P. This training process can be expressed as a problem of finding weight matrix W, bias matrix Θ, and activation function vector f such that:

(20) $$net(P^{\prime },W,\Theta ,f)=P$$

The NN architecture consists of two neurons in both the input and output layers, while the number of neurons and hidden layers are experimentally determined in Section 3.1. The outputs of the calibration stage are: the estimated apparent beacon positions $A_{i}=\left(X_{i},\;Y_{i}\right),\;i=1,\cdots ,m$, and the trained neural network represented as N=(W,Θ,f), where W is the weight matrix, Θ is the bias matrix, and *f* is the vector of activation functions.

The offline calibration stage needs only to be executed once when the positioning system is initially set-up. The time required for this procedure depends mostly on the number of the reference positions in pattern P. The expected accuracy improvement is also based on the number of pattern samples.

**Online stage.** In the online stage, the distances $d_{ij}$ between the beacons and the receiver are measured, and the initial position estimate $r^{\prime }=\left(x^{\prime },\;y^{\prime }\right)$ is provided through NLS solver where the beacon positions $A_{i}=\left(X_{i},\;Y_{i}\right),\;i=1,\cdots ,m$ are set according to the ABPE estimation obtained in the calibration stage. This position estimate $r^{\prime }$ is further improved by setting it as the input of the neural network and acquiring the appropriate output. The NN used in this stage represents the one with the best validation performance obtained within the training process in the offline stage. The output of the network $r^{\prime \prime }=\left(x^{\prime \prime },\;y^{\prime \prime }\right)=net\left(r^{\prime },W,\Theta ,f\right)$ is the corrected estimate for the position of the receiver and is the final output of the hybrid method. By achieving such output, the proposed calibration method is able to predict a more accurate estimate of the receiver position while simultaneously mitigating the systematic error.

The online stage algorithm is integrated into the positioning system driver and is performed whenever a new position measurement is queried. The correction calculation adds a very minor overhead and thus can be implemented in a real-time system. The online stage does not require an involvement of the operator in terms of additional set-up.

## 3. Experimental Results

### 3.1. Experiment 1

The selection of the appropriate learning algorithm, the activation function of neurons, and neural network architecture (the number of layers and the number of neurons in each layer) are significant problems in neural network design. In order to test the performance of the proposed method, experiment 1 was performed for preliminary tuning of the parameters of the neural network. For this experiment, 158 data samples were collected using the UR5 robot-based high-precision reference system described in [34]. The neural networks are trained with the use of 10 different learning algorithms presented in Table 1.

Furthermore, 16 neural network architectures (one-layered, two-layered, three-layered, and four-layered architectures), with different numbers of neurons in each layer, are used in order to find the optimal neural network structure for the current calibration problem. Taking the one-layered architectures as an example, the number of neurons is adopted from minimal 3 to maximal 15. Four two-layered architectures are tested with the number of neurons in the first layer varied from 3 to 5 and in the second layer from 3 to 15. The number of neurons in four three-layered architectures varies from 3 to 5 in the first layer, from 3 to 10 in the second layer, and from 3 to 15 in the third layer. Finally, four different four-layered architectures are also tested; for example, the network architecture represented as 5-5-10-15 means that there are 5 neurons in both the first and second hidden layers, 10 neurons in the third, and 15 neurons in the fourth hidden layer. The list of 16 aforementioned architectures is shown in Table 2.

Another goal of this experiment was to test the effect of employing different activation functions of the neurons to the learning performance of the network. Table 3 shows 12 activation functions (‘logsig’, ‘tansig’, ‘softmax’, ‘radbas’, ‘compet’, ‘tribas’, ‘hardlim’, ‘hardlims’, ‘poslin’, ‘purelin’, ‘satlin’, ‘satlins’) used for tuning the neural networks.

Altogether, 10 learning algorithms are used, with 16 neural network architectures and 12 different activation functions for hidden layers. Therefore, to assess the performance of the proposed approach, the total number of tested neural networks is 10×16×12=1920.

After the preliminary experimental tuning, the learning rate for all neural network architectures is set to 0.01. The training process is stopped when the root mean square error RMSE falls below $10^{-4}$ cm or in the case when the maximum number of iterations (2000) is reached. The experimental runs were repeated 50 times in order to collect data for statistical analysis. The algorithms were developed and experimentally validated in MATLAB environment running on the AMD Ryzen 7 3.8 GHz processor desktop computer with 8 GB of RAM. The input/output pairs (i.e., reference position/measured position) are divided in the following manner: 70% of data were used for training, and 30% of data were used for validation and testing. The accuracy of the network is measured with RMSE, where a lower value of the error indicates better calibration performance.

**Figure 3:**
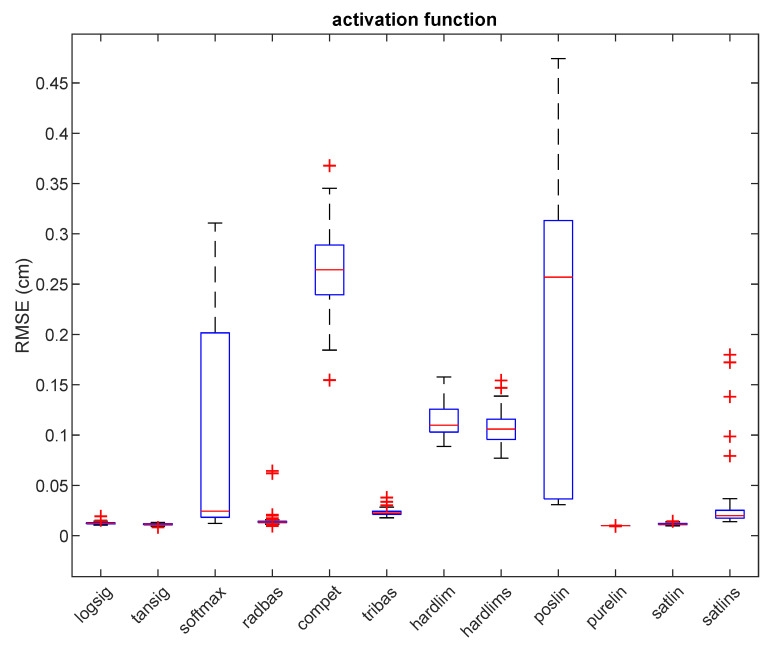
Testing RMSE for 12 different activation functions in experiment 1. Red lines show the median, the blue boxes encompass the 25th and the 75th percentiles, the whiskers represent the range and the plus signs indicate outliers.

Table 3 shows comparative best result for 12 activation functions. These results are based on the series of trials where we have varied the network architecture, learning algorithm, and activation function. Detailed results of these calculations for the case of the ‘purelin’ activation function (lowest average RMSE of 0.99 cm) is shown in Table 4. As can be seen, ‘logsig’, ‘tansig’, ‘softmax’, ‘radbas’, ‘purelin’, and ‘satlin’ activation functions achieve the lowest values of minimum RMSE, and therefore they were chosen to be used in the further experiments (2 and 3). Moreover, for four out of six of the best activation functions, the best RMSE value was achieved with the Levenberg–Marquardt back-propagation algorithm. Figure 3 shows box plots of RMSE after 50 independent trials and reveal that the minimum RMSE is achieved with the ‘purelin’ activation function.

### 3.2. Experiment 2

**Figure 4:**
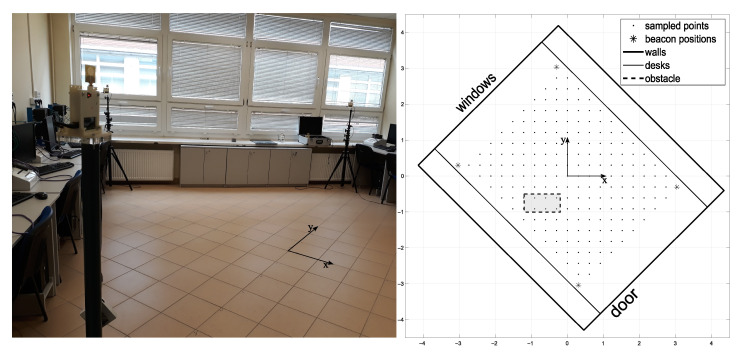
Experimental site.

In the second experiment, the aim was to test the performance of the hybrid NN-ABPE method over real-world datasets. Therefore, experimental data were collected in real-world conditions, i.e., the following set-up measuring system was placed in a 6.5 × 5.5 m empty room. Four anchors were located in positions indicated in Figure 4; measurement positions were taken on every vertex of the square grid with the spacing of 0.30 m.

The collected data were preprocessed by using the ABPE method and were used to train the neural networks. The goal of this experiment was to find the best neural network using the best activation functions obtained in experiment 1 (‘logsig’, ‘tansig’, ‘softmax’, ‘radbas’, ‘purelin’, ‘satlin’) and the best learning algorithm from experiment 1 (Levenberg–Marquardt back-propagation). The other parameters were set as follows: learning rate was adopted as 0.01, the stopping criteria for training were reaching the RMSE of $10^{-4}$ cm or reaching the maximum number of learning iterations (2000); all calculations were repeated 50 times.

**Figure 5:**
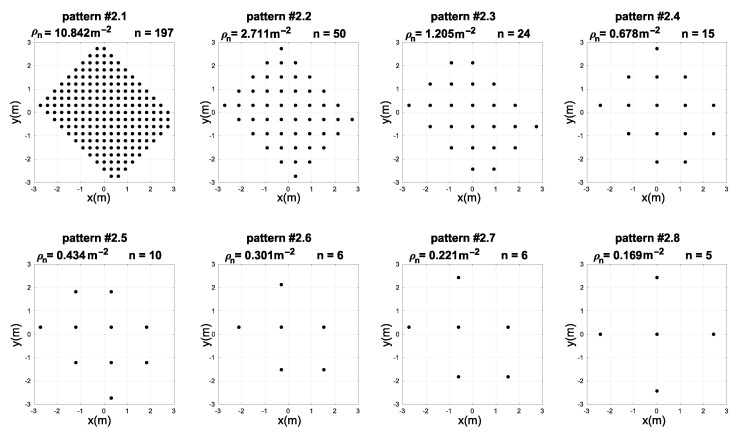
Measuring position patterns with different number of densities $\rho_{n}$ and number of points *n* used for training of the neural networks in experiment 2.

In order to test the usefulness of our proposed method, it is necessary to test how robust the accuracy improvement is, depending on the number and the pattern of the training samples. Typically, uniform sampling patterns of varying sampling densities are used in real conditions. In this experiment, we defined eight sampling patterns, in which the sampling points were selected from the previously collected dataset such as to form uniform grid distributions with varying sampling densities. These sampling patterns, arranged from the highest density (number of samples) to the lowest, are presented in Figure 5. We have compared the accuracy improvement results of the state-of-the-art methods (DQM, ABPE, BSFE) and the different configurations of our proposed hybrid NN-ABPE method for these sampling patterns. The results are shown in Table 5. The improved rate IR is computed as follows (21):

(21) $$IR=\frac{RMSE_{BSFE}-RMSE_{NN-ABPEpurelin}}{RMSE_{BSFE}}\times 100\%$$

As can be seen, the results achieved with the proposed hybrid method indicate a noticeable improvement in calibration accuracy. For seven out of eight tested sampling point distribution patterns, the NN-ABPE method with a ‘purelin’ activation function showed the best result with minimal RMSE. Moreover, the hybrid NN-ABPE method achieves better results when compared with the model based only on NNs (NN-RAW purelin). The overall success calculated by improved rate (IR) ranges from 5.22% (for pattern #2.1) to 41.83% (for pattern #2.6).

**Figure 6:**
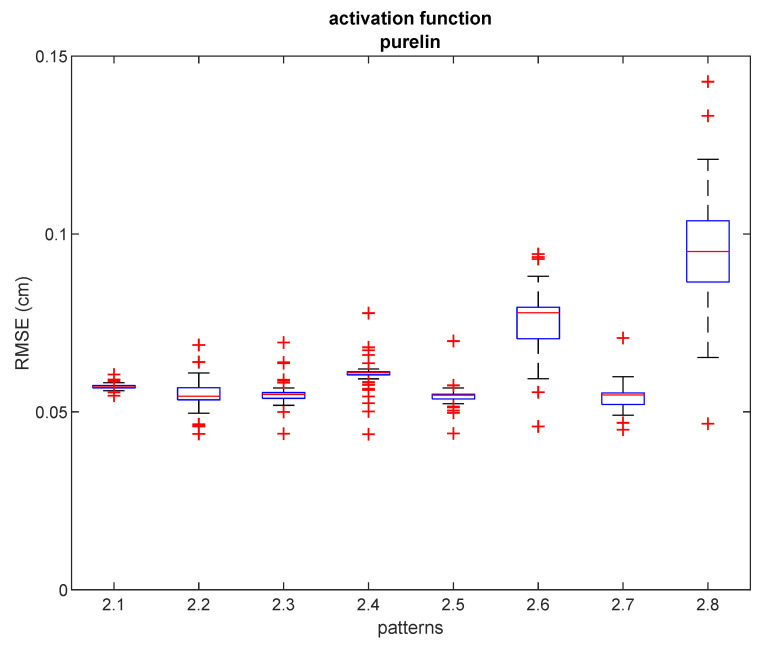
RMSE for ‘purelin’ activation function and different densities in experiment 2. Red lines show the median, the blue boxes encompass the 25th and the 75th percentiles, the whiskers represent the range and the plus signs indicate outliers.

Figure 6 shows box plots of RMSE results after 50 repetitions with the hybrid NN-ABPE approach and a ‘purelin’ activation function. It can be concluded that even if there is a relatively small number of training samples (e.g., six training samples for pattern #2.6 and pattern #2.7, or five training samples for pattern #2.8), the proposed method achieves a high level of accuracy in scenarios where fast set-up time is an essential requirement.

### 3.3. Experiment 3

**Figure 7:**
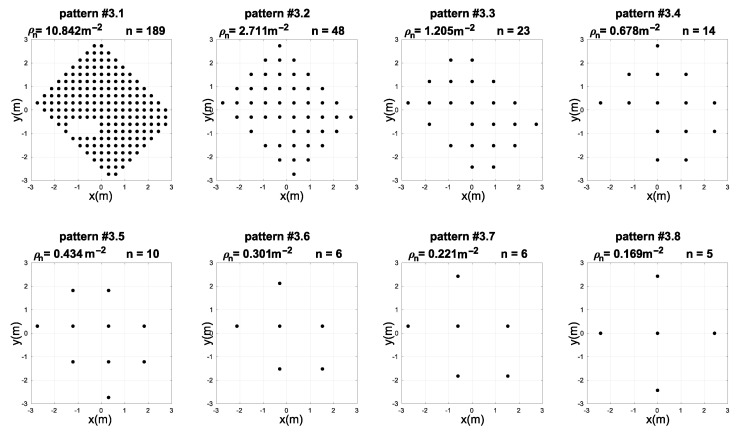
Measuring position patterns with different number densities $\rho_{n}$ and number of points *n* used for training of the neural networks in experiment 3.

The third experiment was performed to test the hybrid NN-ABPE method in a real-world set-up including obstacle. The data set was collected in the same laboratory environment with a single obstacle in the form of a metal cabinet (1 × 0.4 m) placed in the middle of the room. The experimental set-up consists of four anchors, which were located as shown in Figure 4, while the measurement positions were taken on every vertex of the square grid with the spacing of 0.3 m. The obstacle is located in the environment as shown in Figure 4. The addition of the obstacle necessitates a change in the sampling pattern as shown in Figure 7.

The goal of this experiment was to find the best NN architecture while using the best activation function and learning algorithm selected in experiment 1. The other parameters of the neural networks are set as follows: learning rate is adopted as 0.01; the stopping criteria for training is achieving the RMSE of $10^{-4}$ cm or reaching the maximum number of learning iterations (2000). All calculations are repeated 50 times.

Table 6 shows the comparative results of the proposed hybrid NN-ABPE method and the other state-of-the-art calibration methods (DQM, ABPE, BSFE). The experimental results obtained with the proposed hybrid method indicate a noticeable improvement in calibration accuracy. For all eight patterns testing the different uniform distributions (i.e., densities of the positions taken for the training of the neural networks), the NN-ABPE method with a ‘purelin’ activation function showed the best result with minimal RMSE. The IR for all patterns is over 56%; the best-improved rate of 91% is reported for pattern #3.1, while the second-best improvement of 67% is recorded for pattern #3.8.

**Figure 8:**
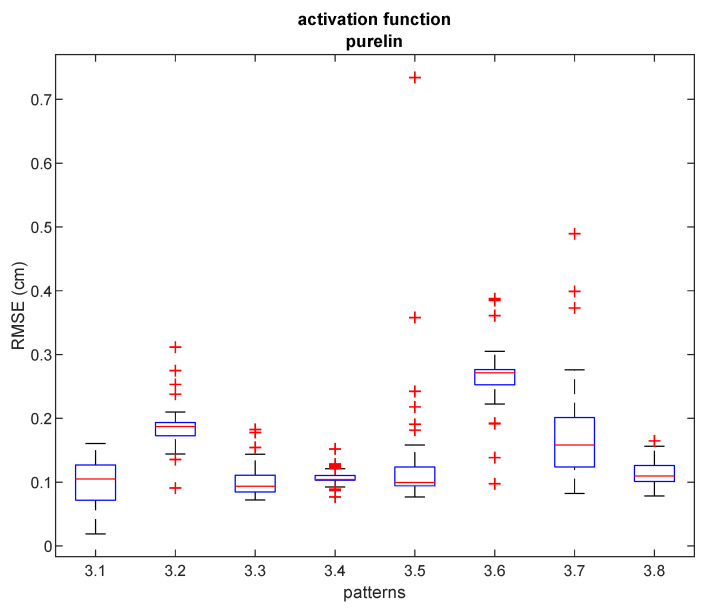
RMSE for ‘purelin’ activation function and different patterns in experiment 3. Red lines show the median, the blue boxes encompass the 25th and the 75th percentiles, the whiskers represent the range and the plus signs indicate outliers.

Box plots of RMSE results after 50 repetitions achieved with hybrid NN-ABPE approach and ‘purelin’ activation function are presented in Figure 8. It can be concluded that even when the obstacles are placed in an indoor environment, the proposed method can obtain a high level of accuracy (RMSE of 1.90 cm) when all data samples are measured. Moreover, in scenarios where the fast set-up time is an essential requirement, experimental results demonstrate that a relatively small number of training samples (i.e., 5–7 measured points) is sufficient to achieve a high level of accuracy; e.g., 10 training samples for pattern #3.5 leads to RMSE of 7.70 cm, while 5 training samples for pattern #3.8 provide RMSE of 8.05 cm.

## 4. Conclusions

In this paper, the authors propose and experimentally evaluate a novel quick set-up calibration method used for accurate compensation of positioning system measurement errors. The proposed hybrid approach is based on back-propagation neural networks trained on data estimated by the Apparent Beacon Position Estimation method (ABPE). The ABPE method determines the so-called apparent beacon positions, which are then used to estimate the receiver position by the NLS method.

Furthermore, the proposed learning mechanism based on neural networks is employed to predict the relation between the reference position and the position obtained through the ABPE method. Different neural network structures, learning algorithms, and activation functions are experimentally evaluated in order to find the optimal solution for the real-world implementation. For fine-tuning purposes, 16 neural network architectures with 10 learning algorithms and 12 different activation functions for hidden layers are investigated in MATLAB environment (1920 networks are tested in total). The results from experiment 1 show that a neural network trained by the Levenberg–Marquardt algorithm and ‘purelin’ activation function provides the overall best performance.

Moreover, in order to show the robustness of the proposed approach, the method is validated in two new experimental studies and compared with the state-of-the-art calibration methods, i.e., Distortion Quadratic Model (DQM), Bias and Scale Factor Estimation (BSFE), and Apparent Beacon Position Estimation (ABPE). The first real-world scenario foresees an indoor environment without obstacles, while the second one considers the measurement of reference positions in a laboratory environment with additional obstacles introduced.

Results from experiments 2 and 3 show that the proposed hybrid NN-ABPE method can predict the location of the target with a high level of accuracy (RMSE of 4.37 cm in experiment 2 with n=15 samples and RMSE of 1.90 cm in experiment 3 with n=189 samples). When it comes to the number of samples necessary to realize a fast set-up procedure, the achieved experimental results demonstrate the superiority of the proposed NN-ABPE method trained with Levenberg–Marquardt backpropagation algorithm and linear activation function ‘purelin’ over the state-of-the-art methods. For the lowest number of samples, it is necessary to have only five reference points, and the proposed method will predict the position of the target object with RMSE of 4.66 cm (experiment 2—without obstacles), which is a 33% improvement over raw results, and RMSE of 8.05 cm (experiment 3—with an added obstacle) with 68% improvement over the raw results.

It is worth noting that both DQM and BSFE methods seem to give slightly better results and thus are more suited to be the first stage of the hybrid method. However, it is important to observe that these methods do require *a priori* knowledge of the beacon positions. Since the ABPE method does not depend on that information, it is a much better candidate when a short set-up time is desired. Thus, we have constructed our hybrid method with the ABPE as the initial step.

Bearing in mind that the choice of the number and the pattern of the sampling points play an essential role in setting up the system, one of the future research directions might be oriented towards a new methodology for the optimal selection of the location of the reference points used in the calibration procedure.

It would also be prudent to establish the level of possible accuracy improvement using the proposed hybrid NN-ABPE method in the range of possible environments by performing multiple additional experiments in real-life industrial scenarios. 

## Figures and Tables

**Table 1:** Summary of learning algorithms used for neural networks development.

No.	Learning Algorithm	Acronym
1	Levenberg–Marquardt back-propagation	LM
2	Bayesian regularization	BR
3	Resilient back-propagation	RP
4	Scaled conjugate gradient back-propagation	SCG
5	Gradient descent back-propagation	GD
6	Gradient descent with momentum back-propagation	GDM
7	Gradient descent with momentum and adaptive learning rule back-propagation	GDMA
8	Powell–Beale conjugate gradient back-propagation	PB
9	Fletcher–Powell conjugate gradient back-propagation	FP
10	Polak–Ribiére conjugate gradient back-propagation	PR

**Table 2:** Neural network architectures.

No.	Architecture	No.	Architecture	
1	3	9	3-3-3	
2	5	10	5-5-5	
3	10	11	3-5-10	
4	15	12	5-10-15	
5	3-3	13	3-3-3-3	
6	5-5	14	5-5-5-5	
7	5-10	15	3-3-10-10	
8	3-15	16	5-5-10-15	

**Table 3:** Best results for 12 activation functions in the experiment 1. Best six activation functions according to minimum RMSE are highlighted. Bold text indicates the best value in the column.

Activation Function	Arch	Alg	RMSE_Best [cm]			
			Max	Min	Median	Average
logsig	10	1	1.93	1.06	1.22	1.24
tansig	6	1	1.31	**0.82**	1.14	1.13
softmax	11	8	31.07	1.21	2.44	8.58
radbas	6	1	6.43	0.95	1.35	1.58
compet	3	3	36.77	15.46	26.42	26.63
tribas	2	9	3.79	1.78	2.25	2.35
hardlim	4	3	15.77	8.88	10.98	11.44
hardlims	4	2	15.43	7.71	10.59	10.66
poslin	15	7	47.41	3.08	25.70	19.11
purelin	9	1	**1.01**	0.93	**0.99**	**0.99**
satlin	7	2	1.43	0.96	1.14	1.16
satlins	11	9	17.98	1.39	1.99	3.21

**Table 4:** Experiment 1 results for ‘purelin; activation function—best, average, and standard deviation for the testing set.

Arch		LM [cm]	BR [cm]	RP [cm]	SCG [cm]	GD [cm]	GDM [cm]	GDMA [cm]	PB [cm]	FP [cm]	PR [cm]
3	Best	0.99	0.98	0.96	0.97	0.96	0.96	0.95	0.97	0.96	0.97
	Ave	1.00	3.31	1.00	1.00	4.15	4.16	1.51	1.00	1.00	1.00
	Std	0.01	3.29	0.03	0.04	5.78	5.81	3.34	0.02	0.02	0.04
5	Best	0.99	0.97	0.97	0.97	0.95	0.95	0.96	0.98	0.97	0.98
	Ave	0.99	2.58	1.02	1.00	1.15	1.14	1.03	1.00	1.00	1.00
	Std	0.01	2.95	0.04	0.03	0.37	0.36	0.05	0.02	0.01	0.01
10	Best	0.99	0.95	0.96	0.96	0.97	0.97	0.93	0.98	0.98	0.97
	Ave	0.99	3.38	1.02	1.00	0.99	0.99	1.05	1.00	1.00	1.00
	Std	0.00	3.97	0.06	0.04	0.02	0.02	0.10	0.02	0.04	0.03
15	Best	0.96	0.97	0.97	0.97	0.98	0.98	0.97	0.97	0.98	0.97
	Ave	1.00	2.48	1.02	1.00	0.99	0.99	1.05	1.00	1.00	1.01
	Std	0.02	2.91	0.04	0.02	0.02	0.02	0.08	0.03	0.03	0.04
3-3	Best	0.99	0.97	0.96	0.97	0.95	0.95	0.95	0.97	0.97	0.98
	Ave	0.99	0.99	1.00	1.00	3.96	5.54	3.97	1.00	1.00	1.02
	Std	0.01	0.01	0.03	0.02	6.87	9.42	9.00	0.02	0.03	0.05
5-5	Best	0.99	0.97	0.93	0.96	0.95	0.96	0.97	0.97	0.96	0.97
	Ave	0.99	0.99	1.00	1.00	1.00	7.79	2.68	1.00	1.00	1.01
	Std	0.00	0.01	0.04	0.04	0.02	16.33	8.23	0.02	0.02	0.05
5-10	Best	0.96	0.97	0.97	0.97	0.98	0.98	0.97	0.97	0.97	0.97
	Ave	0.99	1.65	1.01	1.00	1.47	9.77	1.54	1.00	1.00	1.01
	Std	0.01	4.68	0.04	0.01	3.36	17.28	3.61	0.03	0.03	0.03
3-15	Best	0.99	0.96	0.97	0.96	0.98	0.98	0.97	0.97	0.98	0.98
	Ave	0.99	0.98	1.01	1.01	1.00	12.80	2.53	1.01	1.00	1.00
	Std	0.01	0.01	0.05	0.05	0.02	19.22	6.17	0.04	0.02	0.02
3-3-3	Best	0.93	0.97	0.98	0.97	0.96	0.97	0.95	0.95	0.96	0.97
	Ave	0.99	5.76	1.02	1.01	6.39	9.68	6.88	1.27	1.00	1.01
	Std	0.01	10.34	0.04	0.03	9.12	12.53	12.01	1.93	0.02	0.03
5-5-5	Best	0.99	0.96	0.94	0.99	0.97	0.97	0.98	0.98	0.97	0.97
	Ave	0.99	4.04	1.03	1.00	2.61	17.18	5.89	1.01	1.01	1.00
	Std	0.01	8.35	0.07	0.02	6.51	22.91	12.88	0.04	0.04	0.02
3-5-10	Best	0.96	0.97	0.97	0.96	0.96	0.97	0.94	0.97	0.98	0.97
	Ave	0.99	5.14	1.01	1.01	1.78	26.39	2.93	1.01	1.13	1.00
	Std	0.01	10.49	0.03	0.04	4.21	25.65	7.61	0.04	0.86	0.03
5-10-15	Best	0.99	0.96	0.94	0.97	0.98	0.98	0.93	0.97	0.96	0.97
	Ave	0.99	2.54	1.01	1.00	1.00	30.82	1.02	1.00	1.00	1.01
	Std	0.01	6.19	0.04	0.02	0.02	27.45	0.06	0.01	0.03	0.04
3-3-3-3	Best	0.98	0.97	0.96	0.97	0.94	0.96	0.95	0.94	0.96	0.97
	Ave	1.00	18.11	1.02	1.00	10.02	15.59	14.10	1.01	4.70	1.00
	Std	0.02	13.87	0.04	0.04	13.75	15.33	16.02	0.04	7.76	0.03
5-5-5-5	Best	0.99	0.97	0.97	0.97	0.98	0.98	0.97	0.96	0.94	0.97
	Ave	0.99	17.53	1.01	1.00	1.52	22.82	6.67	1.01	1.16	1.00
	Std	0.01	13.93	0.04	0.02	3.66	24.46	15.89	0.04	0.87	0.03
3-3-10-10	Best	0.99	0.97	0.95	0.95	0.97	0.98	0.96	0.97	0.97	0.97
	Ave	1.00	20.83	1.00	1.01	1.93	35.23	5.39	1.01	1.65	1.02
	Std	0.01	13.54	0.03	0.03	6.56	27.02	10.59	0.05	3.11	0.10
5-5-10-15	Best	0.98	0.97	0.96	0.94	0.98	0.98	0.97	0.97	0.97	0.96
	Ave	0.99	19.39	1.01	1.02	1.00	55.34	2.65	1.01	1.01	1.01
	Std	0.00	13.07	0.03	0.06	0.02	75.14	8.13	0.04	0.04	0.06

**Table 5:** Position RMSE for the different patterns and methods in experiment 2—without obstacles (in [cm]). Best result for each of the patterns is presented in bold.

Pattern	n	Raw [cm]	DQM [cm]	ABPE [cm]	BSFE [cm]	NN—ABPE Logsig [cm]	NN—ABPE Tansig [cm]	NN—ABPE Softmax [cm]	NN—ABPE Radbas [cm]	NN—ABPE Purelin [cm]	NN—RAW Purelin [cm]	NN—ABPE Satlin [cm]	IR [%]
#2.1	197	6.94	5.98	6.29	5.75	5.01	4.66	5.98	5.09	5.45	6.65	**4.11**	5.22
#2.2	50	6.94	6.18	6.38	5.74	5.33	5.29	5.41	5.16	4.38	**4.10**	4.96	23.69
#2.3	24	6.94	6.81	6.33	5.72	4.74	5.17	5.47	5.03	**4.39**	4.79	4.92	23.25
#2.4	15	6.94	6.68	6.72	6.15	5.49	5.61	5.38	5.76	**4.37**	5.06	4.95	28.94
#2.5	10	6.94	7.67	6.34	5.90	5.46	5.21	5.36	5.49	**4.39**	4.95	5.86	25.59
#2.6	6	6.94	10.91	9.60	7.89	7.90	7.33	7.23	10.42	**4.59**	4.73	8.39	41.83
#2.7	6	6.94	8.32	6.40	7.14	5.18	5.26	5.28	7.01	**4.50**	4.89	5.59	36.97
#2.8	5	6.94	8.09	9.39	6.01	9.02	9.56	8.45	38.30	**4.66**	4.75	11.93	22.46

**Table 6:** Position RMSE for the different patterns and methods in experiment 3—with obstacles (in [cm]). Best result for each of the patterns is presented in bold.

Pattern	n	Raw [cm]	DQM [cm]	ABPE [cm]	BSFE [cm]	NN—ABPE Logsig [cm]	NN—ABPE Tansig [cm]	NN—ABPE Softmax [cm]	NN—ABPE Radbas [cm]	NN—ABPE Purelin [cm]	NN—ABPE Satlin [cm]	IR [%]
#3.1	189	25.19	17.09	20.63	21.13	3.93	3.42	5.62	4.42	**1.90**	3.86	91.01
#3.2	48	25.19	17.84	21.51	21.26	15.91	13.76	15.86	17.84	**9.08**	13.21	57.29
#3.3	23	25.19	19.54	20.76	21.99	8.22	8.27	8.30	8.78	**7.22**	8.00	67.17
#3.4	14	25.19	20.18	21.40	22.39	10.74	10.14	10.56	10.96	**7.67**	9.87	65.74
#3.5	10	25.19	18.89	21.03	21.26	10.34	10.41	10.18	10.49	**7.70**	9.52	63.78
#3.6	6	25.19	21.29	26.36	22.32	30.00	32.66	28.74	56.05	**9.74**	29.20	56.36
#3.7	6	25.19	20.34	26.86	22.60	16.29	19.52	17.27	39.13	**8.22**	18.92	63.63
#3.8	5	25.19	23.74	24.32	24.62	11.32	28.08	15.14	62.87	**8.05**	14.42	67.30

## Data Availability

Not applicable.
